# Numerical analysis of flow configuration and channel design for thermoelectric OTEC systems

**DOI:** 10.1038/s41598-025-06415-w

**Published:** 2025-08-05

**Authors:** Chun-I Wu, Wei-Lun Tseng, Bo-Xiang Wang

**Affiliations:** Department of Mechanical and Mechatronic Engineering, National Taiwan Ocean University, Keelung, 20224 Taiwan

**Keywords:** Ocean thermal energy conversion (OTEC), Renewable marine energy systems, Thermoelectric power generation, Bi_2_Te_3_, Thermoelectric materials, Thermoelectric generators, Renewable energy, Energy efficiency, Finite element method, Engineering, Electrical and electronic engineering, Mechanical engineering

## Abstract

This research examines the optimized integration of Bi₂Te₃-based thermoelectric generators (TEGs) in Ocean Thermal Energy Conversion (OTEC) systems, evaluating their performance via detailed numerical analysis. We conducted finite element simulations using COMSOL Multiphysics to analyze thermoelectric generators (TEGs) placed between a warm surface and cold deep seawater channels under different operational conditions. The research examined parallel and counter flow configurations at Reynolds numbers between 3987 and 73,800, with channel heights varying from 0.002 to 0.072 m. Results indicate that Reynolds numbers above 12,000 ensure stable heat supply to TEGs, resulting in a consistent output power of 3.01 W. The optimal net power of 1.45 W was attained at a channel height of 0.002 m, attributed to reduced pump power consumption. A comparative analysis of Bi₂Te₃-based material combinations demonstrated that improved electrical and decreased thermal conductivity notably enhanced system performance. This study offers essential insights for improving the design and implementation of TEG-OTEC systems, especially in offshore contexts where operational efficiency and system durability are critical, thereby contributing to the advancement of sustainable ocean energy technologies.

## Introduction

The global energy landscape faces unprecedented challenges as climate change and depleting fossil fuel reserves necessitate a transition to sustainable energy sources. Ocean Thermal Energy Conversion (OTEC) represents a promising yet underutilized technology that could provide consistent, renewable power generation while addressing these challenges. This research explores the optimization of OTEC systems through the integration of Bi₂Te₃-based thermoelectric generators (TEGs), offering potential solutions to key technological barriers in marine energy harvesting.

### Sustainable energy transition: overcoming fossil fuel challenges

Since the twentieth century, the global energy landscape has faced increasing challenges from depleting fossil fuel reserves and climate change imperatives^1^. While various renewable energy technologies have emerged as alternatives^2,3^, ocean-based energy systems offer unique advantages in addressing these challenges. Unlike intermittent solar and wind power, ocean thermal energy provides consistent baseload power generation capability, making it particularly valuable for coastal and island regions.

Recent technological advances have significantly enhanced the viability of marine energy systems. Notably, breakthroughs in thermoelectric materials have achieved figure-of-merit (ZT) values exceeding 1.5 at room temperature, with materials like SnSe demonstrating ultralow thermal conductivity^4^. These innovations in material science have particular relevance for Ocean Thermal Energy Conversion (OTEC) applications, where efficiency at low-temperature differentials is crucial. The development of hierarchical architectures in thermoelectric materials^5^ has further improved energy conversion efficiency, addressing key challenges in marine energy harvesting.

The integration of advanced materials into ocean energy systems creates robust solutions that capitalize on both the vast thermal resources of the oceans and the maintenance-minimal nature of solid-state devices. This approach offers significant potential for reducing greenhouse gas emissions^6^ while minimizing environmental impact compared to conventional fossil fuel technologies^7^. As global energy demands continue to rise, advancing and implementing sustainable ocean energy technologies has become increasingly critical for combating climate change^8^.

### Exploring ocean thermal energy conversion: principles, research, and applications

This section will delve into the fundamental principles of Ocean Thermal Energy Conversion technology, exploring its various types, recent research advancements, and diverse applications. OTEC systems utilize warm surface seawater and cold deep seawater at an approximate depth of 1000 m as the heat source and cold sink, respectively. These systems harness the natural temperature gradient in the ocean to generate power. Traditional OTEC systems are classified into three types: closed-cycle, open-cycle, and hybrid. Most energy consumption in closed-cycle OTEC is attributed to the operation of pumps for cold seawater, warm seawater, and the working fluid. During operation, the evaporator facilitates thermal energy transfer from the warm seawater to the working fluid, resulting in vaporization. The gaseous working fluid then drives a turbine generator to produce electricity. Subsequently, the condenser uses cold seawater to cool and liquefy the working fluid. The liquid is then returned to the evaporator to repeat the cycle.

OTEC is an emerging renewable energy technology undergoing thorough research. Recent studies have examined different aspects of OTEC, including its performance and the feasibility of combining it with solar energy^9^. Research has also investigated the costs and engineering uncertainties associated with OTEC implementation^10^. Additionally, analyses have explored potential OTEC applications in Japan, such as electricity generation and uranium extraction^11^. Significant work has been conducted on applying solar thermal energy to improve the thermal efficiency of OTEC^12^. Economic factors have also been explored, such as the viability of developing combination plants that simultaneously provide power, freshwater, and mariculture^13^. The Indian 1 MW demonstration OTEC plant has been crucial in advancing OTEC technology^14^.

### Advancing OTEC efficiency: integrating thermoelectric generators

OTEC efficiency can be enhanced by integrating a thermoelectric generator (TEG), which harnesses the temperature difference between warm surface seawater and cold deep seawater to produce electricity. This integration represents a significant advancement in OTEC technology, offering direct energy conversion and system simplification. A key advantage of TEGs is their ability to generate consistent output power, provided a thermal gradient is maintained. The performance of TEGs heavily depends on materials that minimize thermal conductivity while maximizing electrical performance. Recent studies on nanostructured Bi₂Te₃ with semimetal nanoinclusions indicate that nanostructuring techniques can significantly enhance thermoelectric performance by simultaneously boosting power factors and lowering lattice thermal conductivity^15^. This breakthrough provides a strong foundation for optimizing Bi₂Te₃-based materials in OTEC systems, where efficiency at low-temperature gradients is essential. For example, research on oxyselenides has shown that these materials can achieve excellent thermoelectric properties due to their inherently low thermal conductivity^16^. Additional benefits of TEG integration include emission-free operation, long component lifetimes, and no moving parts.

The literature demonstrates extensive research interest in OTEC and thermoelectric-OTEC systems. Burmistrov^17^ and Pourkiaei^18^ both emphasize the promise of thermoelectric technology for harnessing low-grade heat energy. At the same time, Burmistrov highlights the need for further research to improve power density and conversion efficiency. Dogra^19^ and Chopra^20^ comprehensively evaluate OTEC systems. Specifically, Dogra explores different OTEC methodologies, whereas Chopra focuses on developing mathematical models for system performance. Zheng^21^ and Kishore^22^ explore possible thermoelectric technology applications. Zheng analyzes the positive environmental and economic impacts, whereas Kishore proposes an OTEC-based co-generation plant. Wood^23^ presents a comprehensive historical analysis of thermoelectric materials. In contrast, Wei^24^ assesses the efficiency and viability of OTEC organic Rankine cycle systems. Bohn et al.^25^ made a significant advancement in the field by proposing the thermoelectric-OTEC concept and compared it with closed-cycle OTEC in their study. Their research revealed that thermoelectric OTEC has several advantages, including more durable generators with extended lifespans, excellent system reliability due to the lack of moving parts, and improved environmental safety due to pressurized fluids.

Recent studies have further demonstrated the potential of thermoelectric applications in waste heat recovery and renewable energy systems. Sheikholeslami et al.^26^ investigated a photovoltaic cell utilizing hybrid nanofluid and helical fins with thermoelectric generation, achieving significant improvements in electrical efficiency and temperature uniformity. In another study focusing on buildings, Sheikholeslami et al.^27^ examined solar photovoltaic-thermoelectric systems with jet impingement cooling, demonstrating notable enhancements in both electrical and thermal performance. Research by Sheikholeslami et al.^28^ on ternary nanofluid applications in photovoltaic-thermoelectric systems showed promising results for improving system efficiency while considering environmental impacts. These findings complement earlier work on OTEC-TEG integration, particularly in addressing key challenges around heat transfer and system optimization. Additional research by Sheikholeslami et al.^29^ explored the benefits of combining thermoelectric elements with optimized cooling configurations, providing insights into practical implementation strategies.

Integrating Bi_2_Te_3_-based TEGs in OTEC systems offers significant design advantages by reducing pump power requirements and enhancing system reliability. These benefits are particularly critical in offshore environments, where operational stability and low maintenance are essential. Furthermore, Zhao’s ‘panoscopic approach’ demonstrates how structural modifications across multiple scales can significantly enhance material efficiency, offering innovative pathways for improving thermoelectric performance in OTEC applications^30^.

### Optimizing thermoelectric generators: from material innovations to seawater efficiency analysis

Advanced computational methods have become crucial in optimizing TEG performance. Anatychuk et al.^31^ utilized COMSOL finite element software to model and optimize the parameters of thermoelectric couples. Similarly, Yu et al.^32^ used numerical calculations to evaluate the performance of TEGs in recovering waste heat. They examined temperature distributions in both parallel and counter flows. Their simulations demonstrated consistent temperature shifts and enabled refined designs of heat exchangers and thermoelectric couplings to improve waste heat recovery effectiveness in TEGs. In a complementary study, Niu et al.^33^ studied commercially available thermoelectric modules with parallel plate heat exchangers. They aimed to investigate how changes in input temperature, flow rates, and load resistance impact the output power and conversion efficiency of TEGs. Their findings indicate that the hot fluid’s inlet temperature and flow rate substantially influence generated power and conversion efficiency.

The development of advanced Bi_2_Te_3_-based thermoelectric materials continues to be a critical area of research for their potential in near-room temperature applications. Recent works by Shi^34^ and Hong^35^ provide comprehensive reviews of the strategies and progress in enhancing the thermoelectric performance of these materials, including the reduction of lattice thermal conductivity and the use of nanostructures. In particular, Shi^34^ emphasizes the potential of (Bi, Sb)_2_Te_3_ alloys in this regard. Liu^36^ and Zhou^37^ discuss using Bi_2_Te_3_-based materials for mid-temperature power generation and the development of high-performance n-type Bi_2-x_Sb_x_Te_3_, respectively. Tang^38^ and Shu^39^ explore the potential of Bi_2_Te_3_-based thin films and the Mg_3+δ_Sb_x_Bi_2−x_ family as substitutes for the Bi_2_Te_3−x_Se_x_ family. Additionally, Wang^40^ reports on the enhanced thermoelectric properties of Bi_2_(Te_1−x_Se_x_)_3_-based compounds for low-temperature power generation.

The overall system performance of TEGs depends predominantly on the heat transfer rate, which is regulated by various fluid properties, including flow velocity, cross-sectional area of flow channels, and channel geometry. This study addresses critical knowledge gaps in TEG-OTEC integration by examining three key aspects: (1) the impact of cold and warm seawater characteristics on TEG efficiency, specifically investigating how temperature and velocity distributions in the flow channels affect TEG output power and how pump power consumption impacts net power and TEG conversion efficiency; (2) the optimization of channel geometry and flow parameters to maximize system performance while minimizing operational energy losses; and (3) the influence of electrical and thermal conductivities of different thermoelectric materials on TEG output power. Through systematic investigation of these factors, this research aims to establish optimal design parameters for practical TEG-OTEC implementation, particularly for offshore applications where operational efficiency and system durability are essential. Ultimately, these findings contribute to advancing sustainable ocean energy technologies by providing comprehensive guidelines for developing more resilient and efficient marine energy structures.

## Numerical methods

This study employs a combined numerical simulation approach and theoretical calculations to investigate TEG performance in OTEC applications. The numerical analysis uses COMSOL Multiphysics finite element software, complemented by theoretical calculations based on thermoelectric principles and fluid dynamics. The investigation focuses on two distinct configurations: a parallel plate flow configuration and an integrated internal–external channel flow configuration, each analyzed under various operating conditions.

### Modeling and simulation of TEG for OTEC: design and material configuration

**Fig. 1:**
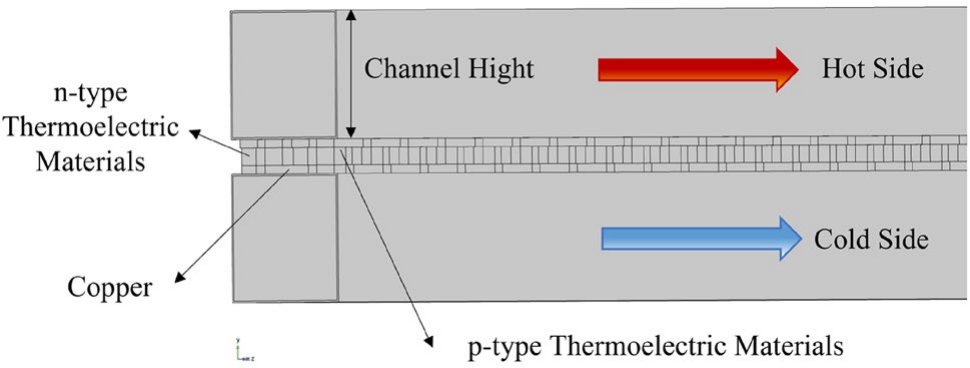
Schematic representation of the channel structure with a height of 0.072 m^44^.

**Fig. 2:**
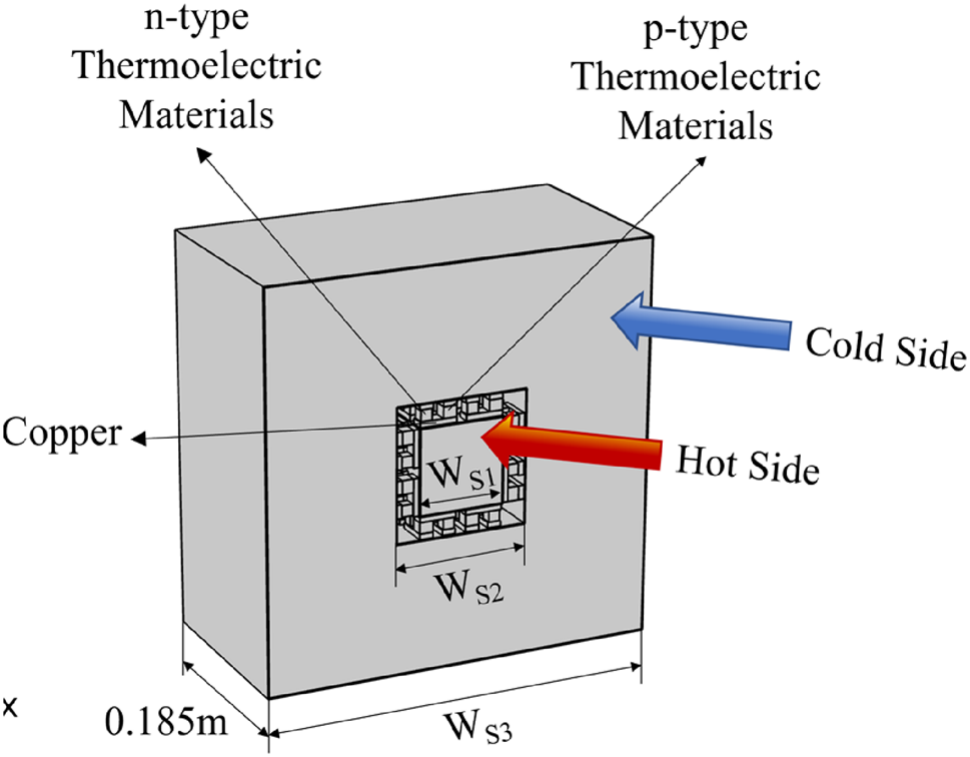
Schematic diagram of the square cross-section flow channel^44^.

This study utilizes COMSOL Multiphysics finite element software to model a TEG for OTEC applications. Two distinct configurations are investigated:

1. Parallel Plate Flow Configuration: The model consists of a TEG between two rectangular flow channels. The upper channel, representing warm surface seawater, acts as the hot side. Meanwhile, the lower channel, representing the cold bottom seawater, serves as the cold side. The warm and cold channels have dimensions of 3.8 m x D m × 0.072 m, where the fluid channel height (D) is varied at 0.002 m, 0.006 m, 0.01 m, 0.018 m, 0.036 m, 0.054 m, and 0.072 m. The TEG connects n-type and p-type thermoelectric materials using copper plates in a series configuration. Simulations examined two different thermoelectric material combinations. The first utilizes 75% Bi_2_Te_3_ + 25% Bi_2_Se_3_ for the n-type and 25% Bi_2_Te_3_ + 75% Sb_2_Te_3_ for the p-type^41^. The second uses Bi_2_Se_0.5_Te_2.5_ for n-type^42^ and (Bi_0.2_Sb_0.8_)_2_Te_3_ for p-type^43^. A single n-type element has dimensions of 0.01 m × 0.01 m × 0.01 m, while a single p-type element has dimensions of 0.012 m × 0.01 m × 0.012 m. The copper plates are 0.03 m × 0.005 m × 0.012 m^41^. There are 400 thermocouple pairs in total. Figure 1 shows the 0.072 m elevation flow channel configuration.
2. Integrated Internal–External Channel Flow Configuration: In this study, the thermoelectric system is integrated into the flow channels, with the thermoelectric modules positioned between an internal flow channel (carrying warm surface seawater) and an external flow channel. The analysis investigates the impact of different volumetric flow rates on the thermoelectric system while maintaining a fixed cross-sectional area, as depicted in Fig. 2.

For the square cross-section channels, the internal flow channel side lengths ${\text{W}}_{\text{S},1}$ were set at 0.075 m, 0.1 m, 0.15 m, and 0.2 m. The external channel cross-sectional area was calculated as ${{\text{W}}_{\text{S},3}}^{2}-{{\text{W}}_{\text{S},2}}^{2}$ m^2^, where the side length of the external channel ${\text{W}}_{\text{S},3}=0.35\text{ m}$, and ${\text{W}}_{\text{S},2}=$ values were set to 0.119 m, 0.144 m, 0.192 m, and 0.244 m.

### Governing equations and assumptions for thermoelectric system simulation

#### Governing equations

The numerical analysis is based on the following assumptions:

- The fluid in the input channel is steady, fully developed, and incompressible.
- The flow channel is thermally insulated, with radiation and convection effects around the channel being disregarded.
- The electrical and thermal resistances at the contact surfaces of the TEG materials are neglected.
- Thermal losses between the heat exchanger and the thermoelectric module are ignored.
- The fluid-solid interface is considered a no-slip boundary.
- Within the TEG module, the leads of the first set of thermocouples are grounded, while all other boundaries of the TEG are set as electrically insulated.

Mass Conservation Equation^45,46^:

1 $$\rho \nabla \cdot u=0.$$

Navier–Stokes Equation:

2 $$\rho \left(u\cdot \nabla \right)u=-\nabla \text{p}+\mu {\nabla }^{2}u.$$

Energy Conservation Equation:

3 $$\rho {c}_{p}u\cdot \nabla \text{T}+\nabla \cdot q=Q.$$

In this context, $\rho ,{c}_{p}$,$\mu$ represent the fluid’s density, specific heat, and viscosity coefficient, respectively; p denotes pressure; $u$ is the velocity vector; T represents temperature. Under thermal insulation, $q$ denotes the heat flux, expressed as $q={k}_{f}\nabla \text{T}$, where ${k}_{f}$ is the fluid’s thermal conductivity; $Q$ represents an internal heat source. The temperature of the fluid can be determined by applying the mass conservation equation, momentum equation (Navier–Stokes equation), and energy conservation equation.

The Heat Conduction Equation for Solids is as follows:

4 $$\nabla \cdot \left({k}_{s}\nabla \text{T}\right)=0.$$

Here, ${k}_{s}$ represents the thermal conductivity of the material. For thermoelectric materials, the energy conservation equation is outlined in references^47–49^:

5 $$\nabla \cdot {q}_{\text{TE}}={Q}_{\text{Joule}}.$$

The Charge Conservation Equation is as follows:

6 $$\nabla \cdot J=0.$$

Within this framework, ${q}_{\text{TE}}$ denotes the heat flux through the thermoelectric material; ${Q}_{\text{Joule}}$ represents the Joule heating generated by the electric current passing through the thermoelectric material, calculated as ${Q}_{\text{Joule}}={J}^{2}/\sigma$, where $J$ is the current density. This is complemented by the thermoelectric constitutive equations referenced in^47–49^:

7 $${q}_{\text{TE}}=STJ-k\nabla T,$$

8 $$J=\upsigma \left(E-S\nabla T\right).$$

Herein, $S,\sigma$,$k$ represent the Seebeck coefficient, electrical conductivity, and thermal conductivity of the thermoelectric material, respectively;$E$ denotes the electric field, which can be expressed as the gradient of electric potential, $-\nabla V$. Consequently, the second constitutive equation can be reformulated as follows:

9 $$J=-\upsigma \left(\nabla V+S\nabla T\right).$$

By incorporating the thermoelectric constitutive equations into the energy conservation and charge conservation equations, one can derive:

10 $$\nabla \cdot \left(STJ\right)-\nabla \cdot \left(k\nabla T\right)=\frac{{J}^{2}}{\sigma },$$

11 $$\nabla \cdot \left(\upsigma \nabla V\right)+\nabla \cdot \left(\upsigma S\nabla T\right)=0.$$

These coupled equations establish the relationship between electric potential and temperature distributions. They determine the temperature and electric potential distributions within the thermoelectric material for specified current conditions and govern the material’s electric potential and current distribution under prescribed temperature conditions.

The heat at the hot end ${Q}_{H}$ and the cold end ${Q}_{C}$ comprises the output power generated by the thermoelectric effect ${P}_{TE}$, the Joule heat produced by the thermoelectric materials ${Q}_{Joule}$, and the conductive heat transferred from the hot end interface to the cold end interface ${Q}_{Cond}$. The equations representing these relationships are as follows:

12 $${Q}_{H}=N\left[S\overline{{T }_{h}}I-\frac{1}{2}{I}^{2}{R}_{TE}+K\left(\overline{{T }_{h}}-\overline{{T }_{c}}\right)\right],$$

13 $${Q}_{C}=N\left[S\overline{{T }_{c}}I+\frac{1}{2}{I}^{2}{R}_{TE}+K\left(\overline{{T }_{h}}-\overline{{T }_{c}}\right)\right],$$

where $N$ represents the number of thermocouples; $S\overline{{T }_{h}}I$ and $S\overline{{T }_{c}}I$ correspond to the output power at the hot end and the cold end, respectively; ${I}^{2}{R}_{TE}$ represents the Joule heat generated by the thermoelectric material; $K\left(\overline{{T }_{h}}-\overline{{T }_{c}}\right)$ is the conductive heat transferred from the hot end interface to the cold end interface. The output power *P* of the TEG can be expressed as^50–52^:

14 $$P={Q}_{H}-{Q}_{C}.$$

The conversion efficiency *η* of the TEG can be expressed as:

15 $$\eta =\frac{P}{{Q}_{H}}.$$

#### Pump power analysis

The fluid pipeline typically consists of straight pipes, bends, and various components, including multiple valves, all contributing to losses in fluid flow. The head loss in the pipeline, accounting for both major losses ${h}_{major}$ and minor losses ${h}_{minor}$, is described as follows:

16 $${h}_{L}={h}_{major}+{h}_{minor}.$$

In pipelines, the energy loss caused by the surface roughness of the straight sections of the pipe is referred to as the major loss, ${h}_{major}$, which can be expressed as:

17 $${h}_{major}=f\frac{{\ell}}{{D}_{h}}\frac{{V}^{2}}{2g}.$$

Herein, $f$ represents the friction factor, ${\ell}$ is the length of the flow channel, ${D}_{h}$ is the hydraulic diameter of the channel, $v$ is the average velocity of the fluid, and $g$ is the acceleration due to gravity.

Minor losses ${h}_{minor}$ primarily occur in non-linear segments of the pipeline and result in energy loss. These losses occur in elements such as bends, valves, inlets, and outlets, or changes in the cross-sectional area of the pipeline and can be expressed as follows:

18 $${h}_{minor}={K}_{L}\frac{{V}^{2}}{2g}.$$

Herein, ${K}_{L}$ represents the loss coefficient, $v$ is the average velocity of the fluid, and $g$ is the acceleration due to gravity. This study conducts analyses under the conditions of steady-state non-viscous fluid flow. For incompressible flow between any two points, 1 and 2, along a streamline, the flow can be described using the Bernoulli Equation:

19 $$\frac{{P}_{1}}{\gamma }+{\alpha }_{1}\frac{{V}_{1}^{2}}{2g}+{z}_{1}+{h}_{P}=\frac{{P}_{2}}{\gamma }+{\alpha }_{2}\frac{{V}_{2}^{2}}{2g}+{z}_{2}+{h}_{L}.$$

Herein, ${P}_{1}$ and ${P}_{2}$ represent the pressures at positions 1 and 2, respectively; ${V}_{1}$ and ${V}_{2}$ are the velocities at positions 1 and 2, respectively; $\gamma$ denotes the specific weight of the fluid; ${\alpha }_{1}$ and ${\alpha }_{2}$ are the kinetic energy correction factors; $g$ is the acceleration due to gravity; ${z}_{1}$ and ${z}_{2}$ are the elevations at points 1 and 2, respectively; ${h}_{P}$ is the pump head; and ${h}_{L}$ represents the head loss.

The pump power can be expressed as follows:

20 $${W}_{P}=\frac{\dot{m}{h}_{P}g}{\eta },$$

where $\dot{m}$ is the mass flow rate, ${h}_{P}$ is the pump head, $\eta$ represents the pump output efficiency, and $g$ is the acceleration due to gravity.

### Model validation

**Fig. 3:**
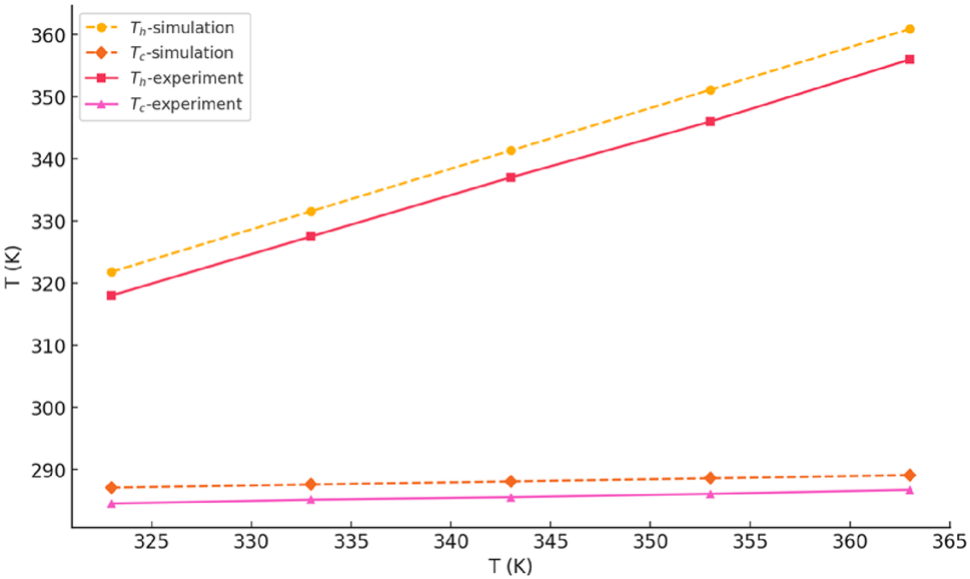
Temperature distribution at the hot and cold ends of the TEG: a comparison of experimental measurements^53^ and simulation results.

To ensure the reliability of the simulations used in this study, our simulation results were benchmarked against the experimental measurements reported by Li et al.^53^. The operating conditions were set such that the flow rates of both heat exchangers were maintained at 1.3 m/s. The fluid temperature entering the cold-side heat exchanger was fixed at 285 K, while the fluid temperature entering the hot-side heat exchanger varied between 323 and 363 K, with simulations conducted at intervals of 10 K. The results indicate the relative error between the simulations conducted at intervals of 10 K. Figure 3 shows the relative error between the simulated average temperatures of the TEG’s hot and cold sides and Li et al.’s experimental data^53^ is 1.1%.

## Results and discussions

This study utilized COMSOL Multiphysics finite element software to simulate a TEG between warm surface seawater and cold deep seawater flow channels. The 3.8-m-long, 0.072-m-wide channels comprised 400 thermoelectric units in total. A parallel-plate heat exchanger established the thermoelectric system model. The researchers examined how different Reynolds numbers (Re) impacted temperature distribution in parallel and counter flows by varying channel heights. They analyzed how heat quantities at the hot (Q_h_) and cold (Q_c_) ends affected TEG output power (P_out_). Additionally, they investigated how pump power consumption influences output power across channel heights. Furthermore, the study examined how alternative thermoelectric materials and cold end temperatures impacted power generation, evaluating how material properties and temperature differentials affect thermoelectric system performance.

### Effects of Reynolds number and channel height on TEG performance in OTEC systems

This study utilized variations in flow channel height to investigate the effects of Reynolds number (Re) on temperature distribution, the heat quantity at both the hot and cold ends of the TEG, and the TEG’s output power under both parallel and counter flow conditions. The flow channel heights (D) were set at 0.002 m, 0.006 m, 0.010 m, 0.018 m, 0.036 m, 0.054 m, and 0.072 m. These corresponded to Reynolds numbers (Re) of 3987, 11,275, 18,040, 29,848, 49,200, 63,253, and 73,800, respectively, as shown in Table 1.

**Table 1:** The correspondence of hydraulic diameter and Reynolds number to channel height.

Channel height (m)	Hydraulic diameter (m)	Reynold number
0.002	3.89 × 10^–3^	3.987 × 10^3^
0.006	1.11 × 10^–2^	1.1275 × 10^4^
0.010	1.76 × 10^–2^	1.8040 × 10^4^
0.018	2.91 × 10^–2^	2.9848 × 10^4^
0.036	4.8 × 10^–2^	4.9200 × 10^4^
0.054	6.17 × 10^–2^	6.3253 × 10^4^
0.072	7.2 × 10^–2^	7.3800 × 10^4^

**Fig. 4:**
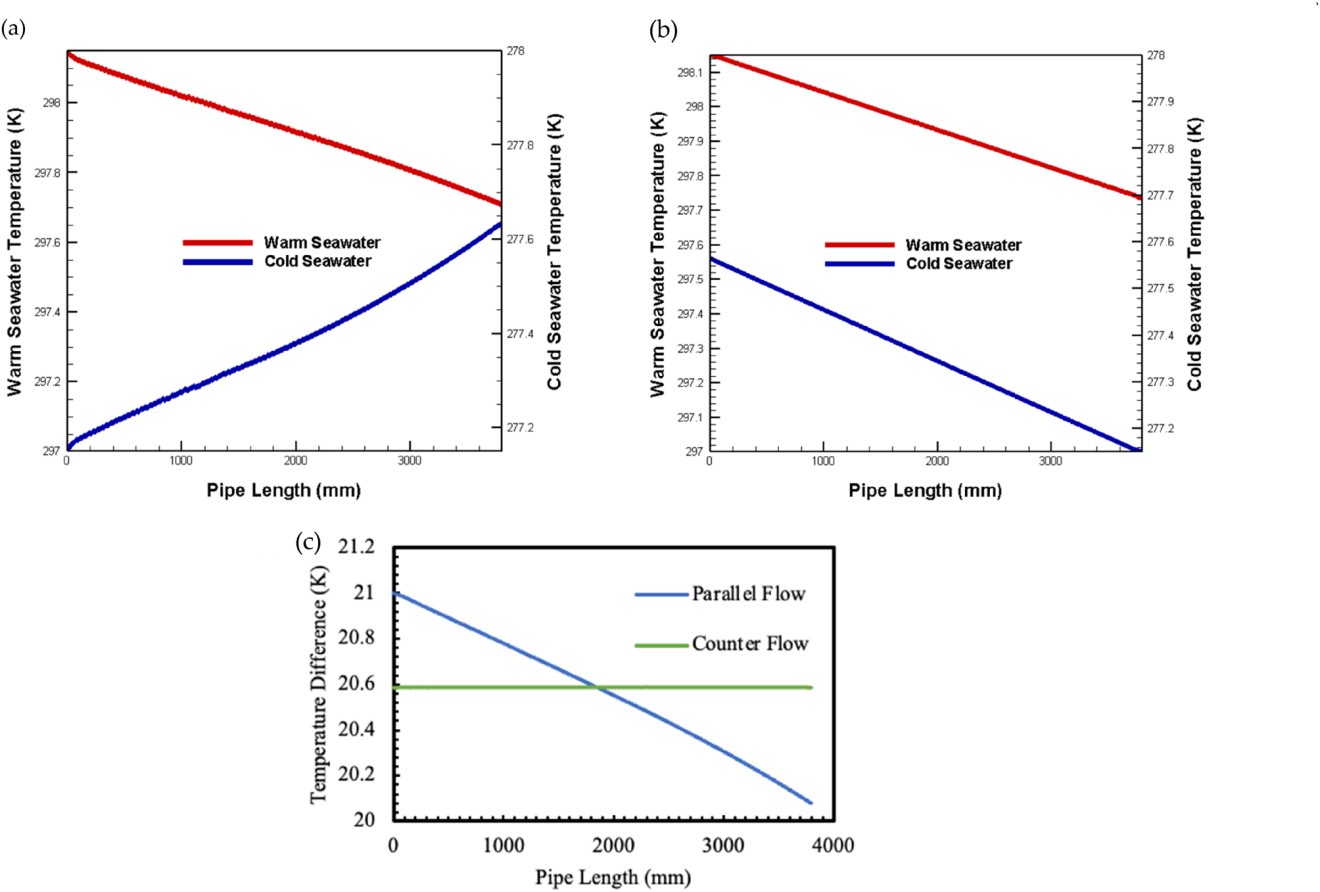
Trend diagram illustrating the relationship between channel length and temperature distribution at a Reynolds number of 3987 (**a**) parallel flows, (**b**) counter flows, (**c**) temperature difference^44^.

The thermal behavior analysis in Fig. 4 reveals the complex interplay between flow dynamics and heat transfer through temperature distributions of warm surface and cold deep seawater along the channel length. For the case of the 0.002 m-tall channel (Re = 3987), both parallel and counter flows were analyzed with inlet temperatures fixed at Tw = 298 K for warm seawater and Tc = 277 K for cold, with an inlet velocity of one m/s (Vin). This configuration was selected to examine heat transfer behavior in the transitional regime between laminar and turbulent flow, where thermal boundary layer development significantly influences heat transfer efficiency. The relatively low Reynolds number, in this case, results in a thicker thermal boundary layer, which affects the temperature gradient between the fluid and channel walls.

The nearly linear temperature changes observed along the flow path indicate steady-state heat transfer conditions, where the thermal resistance between the fluid and TEG remains relatively constant. This behavior is particularly significant for OTEC applications as it suggests predictable and manageable thermal conditions for the TEG operation. The linear profile also implies that the heat transfer coefficient remains relatively stable throughout the channel length despite the developing thermal boundary layer—a characteristic that simplifies system design and control strategies.

The data indicates decreasing warm water and increasing cold water temperatures along the channel. Both fluids display nearly linear temperature changes along the flow path. This behavior suggests that steady heat transfer between the fluids and TEG enables uniform TEG output power, as evidenced by the constant gradient simulation results.

The observed fluctuation in fluid temperatures results in proportional alterations in the temperature differential ∆T between the high and low ends of the TEG. Figure 4c illustrates the distribution of temperature differences in the TEG’s parallel and counter flows. The temperature differential ∆T in the parallel flow shows notable variations in fluid inlet temperature. It decreases as the flow channel length rises, indicating a reduction in the temperature gap between the hot and cold ends. In contrast, the temperature difference in the counter flows shows reduced fluctuations as the channel length increases, enabling the installation of larger channels for TEGs in this model. When the channel length is the same, the average temperature differences in the parallel and counter flows are nearly the same, leading to comparable output powers for both models.

**Fig. 5:**
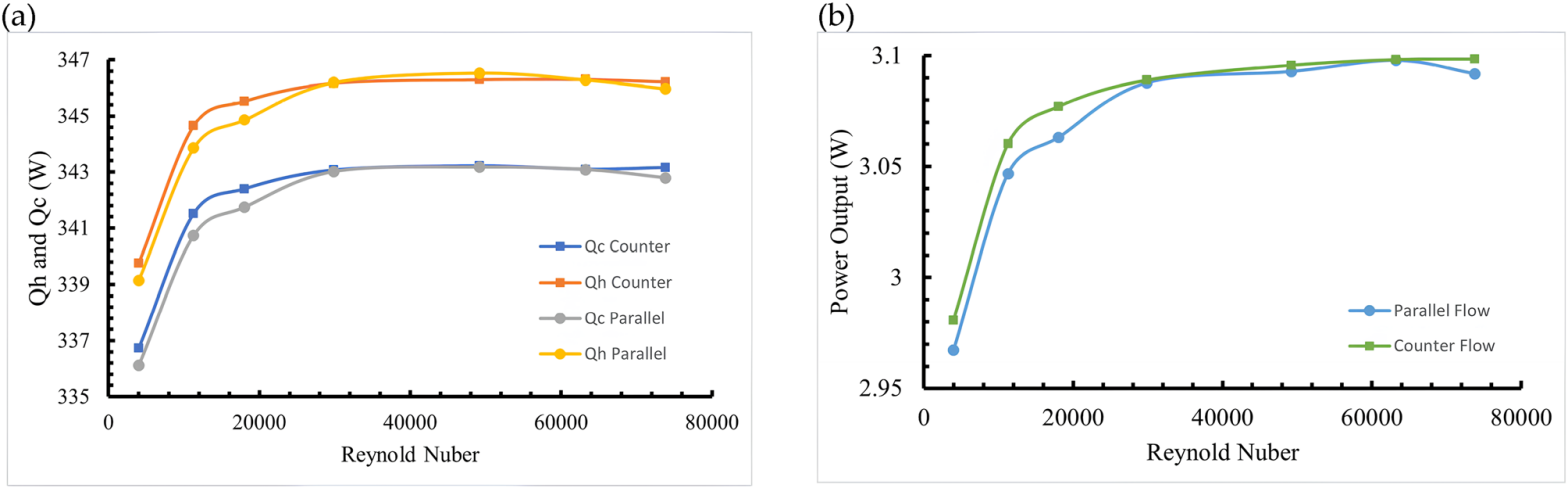
(**a**) Comparative analysis of heat quantities at the hot and cold ends for parallel and counter flows. (**b**) Comparison of output power for parallel and counter flows across various Reynolds numbers^44^.

This study examines the influence of changing Reynolds numbers on the power output of TEGs at varying flow channel heights. Variations in the channel height modify the thermal energy contained in the fluid, thereby impacting the output power of the TEG. Figure 5a depicts the heat amounts (*Q*_*h*_ and *Q*_*c*_) at the hot and cold end for parallel and counter flows at different Reynolds numbers. For Reynolds numbers (Re) below 12,000, the heat available for the TEG increases as the Reynolds number grows. This results in a progressive output power increase for parallel and counter flows models. Nevertheless, when the Reynolds number surpasses 12,000, the heightened channel height facilitates an ample and consistent heat delivery to the TEG. The computed thermal energy at the hot end (*Q*_*h*_) is roughly 345 W, while the thermal energy at the cold end (*Q*_*c*_) is around 342 W, with no disparity in the heat transfer between parallel and counter flows.

The TEG output power (*P*_*out*_) is directly influenced by the heat amounts at the hot end (*Q*_*h*_) and the cold end (*Q*_*c*_). When the Reynolds number (Re) is below 12,000, parallel and counter flows models exhibit the lowest heat quantities at the hot and cold ends of the TEG. This leads to a higher temperature variance in the flow channels compared to models with Re > 12,000. The output power at a Reynolds number of 3987 is the minimum.

The relationship between Reynolds number and TEG performance demonstrates a critical threshold behavior that fundamentally affects system efficiency. As the Reynolds number increases, we observe a corresponding enhancement in TEG output power in both parallel and counter flows, attributed to two key mechanisms:

1. Enhanced Mixing Effects: At Reynolds numbers above 12,000, the flow transitions into a fully turbulent regime, characterized by improved cross-sectional mixing and reduced thermal boundary layer thickness. This enhancement in fluid mixing significantly improves heat transfer coefficients, as turbulent eddies facilitate more efficient thermal energy transport between the fluid and channel walls.
2. Thermal Boundary Layer Development: The reduction in thermal boundary layer thickness with increasing Reynolds number decreases thermal resistance between the fluid and TEG surface. This phenomenon explains why the heat transfer rate stabilizes above Re = 12,000—the boundary layer has reached its minimum effective thickness for the given geometry.

These mechanisms result in a steady heat exchange rate of approximately 345 W at the hot end (Qh) and 342 W at the cold end (Qc), with the difference representing the energy converted to electrical power. This stability in heat transfer rates is crucial for practical OTEC applications, as it ensures consistent power output and simplifies control systems.

As a result, the output power is fixed at roughly 3.01 W. Channel height emerges as a critical design parameter that substantially impacts system performance through multiple mechanisms:

1. Heat Transfer Area Effects: The channel height directly influences the available heat transfer surface area between the fluid and TEG. Our analysis reveals that increasing channel height initially improves heat transfer by providing greater contact area, but this benefit diminishes due to:
  Reduced fluid velocity for a given mass flow rate
  Increased thermal resistance in the fluid bulk
  Development of temperature stratification in larger channels
2. Flow Distribution Impact: The relationship between channel height and flow distribution demonstrates that:
  Smaller channels (D = 0.002 m) promote more uniform flow distribution and better thermal contact
  Larger channels show increased temperature stratification, reducing effective heat transfer
  The optimal channel height balances these competing effects while minimizing pumping power requirements
3. Performance Integration: The combined effects of channel height and Reynolds number on system performance can be understood through the following mechanisms:
  At low Reynolds numbers (Re < 12,000), channel height predominantly affects thermal boundary layer development
  At higher Reynolds numbers (Re > 12,000), the impact of channel height shifts toward flow distribution and pumping power considerations
  The optimal configuration achieves maximum heat transfer while minimizing parasitic losses

This integrated understanding of channel height effects provides crucial guidance for practical OTEC system design, particularly in optimizing the trade-off between heat transfer performance and pumping power requirements. Nevertheless, as the Reynolds number continues to rise, the growth rate in output power decreases, as depicted in Fig. 5b.

The analysis of Reynolds number variations and channel height directly informs the design of OTEC systems by identifying optimal configurations for heat transfer and energy efficiency. These findings provide guidelines for minimizing pump power and maximizing net output, which is critical for OTEC deployment, where operational cost and energy efficiency are primary concerns in ocean engineering.

The simulation results demonstrated that the temperature change exhibits a nearly linear profile when surface warm seawater and deep cold seawater flow through the channels at fixed temperatures and velocities. This linear behavior confirms stable heat exchange between the fluid and TEG. Furthermore, the analysis revealed a critical Reynolds number threshold at Re = 12,000, below which both parallel and counter flows provide lower heat to the TEG. Above this threshold, the system maintains sufficient and stable heat transfer, leading to stabilized output power of 3.01 W.

### Analyzing pump power and channel dimensions in thermoelectric system efficiency

**Fig. 6:**
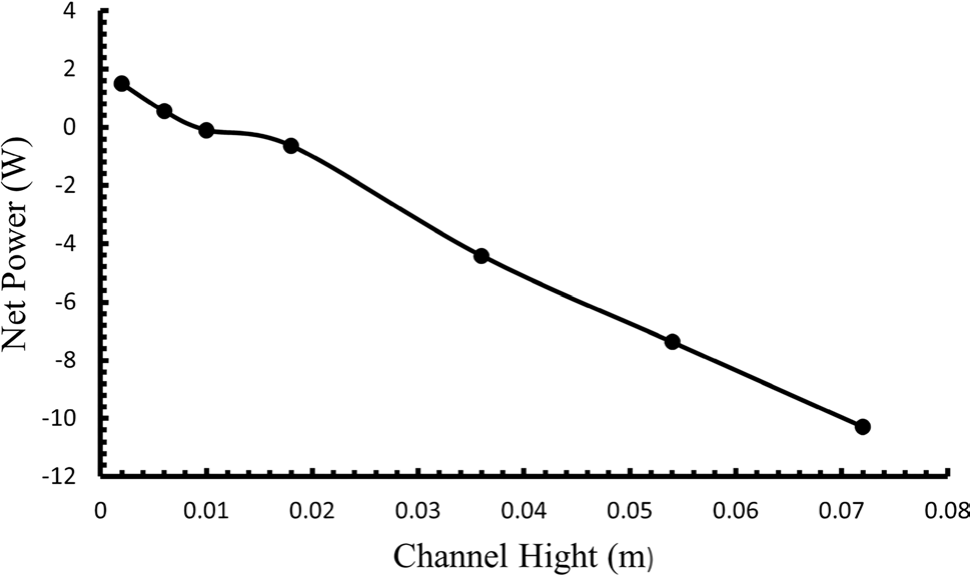
Variation of net power output across various channel heights^44^.

The thermoelectric system’s overall efficiency depends critically on the balance between power generation and parasitic losses, particularly pump power consumption. Figure 6 illustrates the relationship between channel height and net power production, accounting for both TEG output and required pump power. Our analysis reveals the complex relationship between channel geometry, flow parameters, and system efficiency through several key mechanisms:

1. Pump Power Requirements:
  The relationship between pump power and channel height follows a non-linear trend due to:
    Increased fluid volume requiring circulation in larger channels
    Changes in flow resistance characteristics
    Variations in pressure drop across the system length
  The analysis incorporates both major losses (due to wall friction) and minor losses (from inlet/outlet effects), with loss coefficients *K*_*L,in*_ = 0.5 and *K*_*L,out*_ = 1.0
2. Channel Height Optimization:
  At constant velocity, our results demonstrate that while larger channels (D > 0.006 m) improve TEG output power, the optimal net power occurs at D = 0.002 m. This seemingly counterintuitive result can be explained by three primary factors:
  Minimized Pumping Losses: Smaller channels reduce the total fluid volume requiring circulation, significantly decreasing pump power consumption
  Enhanced Heat Transfer Efficiency: Despite reduced cross-sectional area, smaller channels maintain effective heat transfer through:
    Improved thermal boundary layer characteristics
    More uniform temperature distribution
  System Integration Effects: The combination of reduced pumping losses and maintained heat transfer efficiency results in maximum net power production of Pnet = 1.45 W
3. Critical Threshold Analysis:
  The identification of a critical channel height threshold at 0.01 m, above which net power becomes negative, provides crucial design guidance:
  This threshold represents the point where pumping power requirements exceed TEG power generation
  The relationship highlights the importance of compact design in practical OTEC systems
  The finding suggests that multiple parallel small channels might be more effective than fewer large channels

This comprehensive understanding of pump power effects and channel optimization provides essential insights for practical OTEC system design, particularly in offshore applications where system efficiency directly impacts economic viability.

### Comparative analysis of thermoelectric materials and their impact on performance

**Fig. 7:**
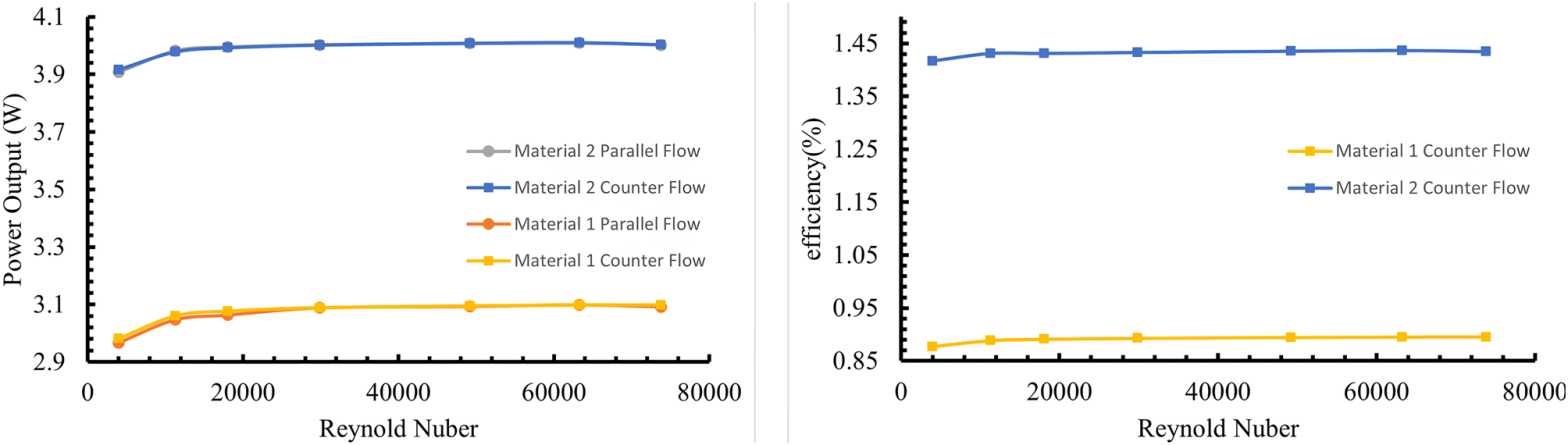
Comparison of (**a**) output power and (**b**) conversion efficiency of different thermoelectric materials in parallel and counter flows (Material 1— n-type: 75% Bismuth Telluride (Bi_2_Te_3_), 25% Bismuth Selenide (Bi_2_Se_3_); p-type: 25% Bi_2_Te_3_, 75% Antimony Telluride (Sb_2_Te_3_); Material 2—n-type: Bismuth Selenide Telluride (Bi_2_Se_0.5_Te_2.5_), p-type: Bismuth Antimony Telluride ((Bi_0.2_Sb_0.8_)_2_Te_3_))^44^.

Thermoelectric performance depends on the Seebeck coefficient and thermal and electrical conductivity. The dimensionless figure of merit (ZT) commonly evaluates materials. Emerging materials, such as high-entropy stabilized chalcogenides, have notably improved thermoelectric performance. Such advancements indicate the potential for future studies to explore alternatives that could efficiently complement or surpass current Bi₂Te₃-based TEG systems^54^. This study examined two distinct thermoelectrics: Material 1, with 75% Bi_2_Te_3_ + 25% Bi_2_Se_3_ as n-type and 25% Bi_2_Te_3_ + 75% Sb_2_Te_3_ as p-type; and Material 2, with Bi_2_Se_0.5_Te_2.5_ (n-type) and (Bi_0.2_Sb_0.8_)_2_Te_3_ (p-type). Figure 7 shows ~ 1 W higher output power for Material 2, which has superior properties, versus Material 1 for parallel and counter flows. Owing to its higher electrical and lower thermal conductivity, Material 2 also surpassed Material 1 in conversion efficiency by 0.5% in the counter-current model (Fig. 7), a trend also seen with parallel flows. Thus, thermoelectrics with improved performance traits increase output power and efficiency.

The selection and optimization of thermoelectric materials represent a crucial aspect of TEG-OTEC system design, with performance characteristics fundamentally linked to three key material properties: the Seebeck coefficient, thermal conductivity, and electrical conductivity. Our comparative analysis of different material combinations reveals several significant insights:

1. Material Performance Mechanisms:
  The superior performance of Material 2 (Bi₂Se₀.₅Te₂.₅/[Bi₀.₂Sb₀.₈]₂Te₃) compared to Material 1 can be attributed to:
  Enhanced Electrical Transport: Higher electrical conductivity reduces internal resistance losses
  Optimized Thermal Properties: Lower thermal conductivity maintains larger temperature gradients
  Improved Power Factor: Better balance between Seebeck coefficient and electrical conductivity
2. Flow Configuration Effects:
  The interaction between material properties and flow configurations reveals that:
  Counter-flow arrangements show particular sensitivity to material selection, with Material 2 achieving 0.5% higher conversion efficiency
  The performance advantage of Material 2 persists across all Reynolds numbers, indicating robust material superiority
  The magnitude of performance improvement varies with flow conditions, suggesting opportunities for optimization
3. Performance Integration:
  The comprehensive analysis demonstrates that:
  Material selection impacts extend beyond simple efficiency metrics
  System-level considerations must include:
    Long-term stability under thermal cycling
    Material cost and availability
    Manufacturing complexity
  The ~ 1 W power output improvement observed with Material 2 represents a significant advancement in OTEC system performance

These findings provide crucial guidance for future material development and system design optimization in TEG-OTEC applications.

### Impact of temperature differential on output power and efficiency in thermoelectric systems

The relationship between temperature differential and thermoelectric performance is fundamental to OTEC system optimization. This study analyzed both power output and efficiency using consistent materials while varying the temperature gradient, the primary driver of thermoelectric performance. The temperature differential’s impact operates through several key mechanisms:

First, a larger temperature gradient enhances the Seebeck effect, which governs the conversion of thermal to electrical energy. This enhancement occurs because the Seebeck effect generates an electromotive force proportional to the temperature difference between the hot and cold junctions. The open-circuit voltage (*V*_*OC*_) follows the relationship:

21 $${V}_{OC}=\left({S}_{p}-{S}_{n}\right)\times \left({T}_{H}-{T}_{C}\right)\times N,$$

where *V*_*OC*_ is Open-circuit voltage (V); *S*_*p*_ is Seebeck coefficient of the p-type thermoelectric material (V/K); *S*_*n*_ is Seebeck coefficient of the n-type thermoelectric material (V/K); *T*_*H*_ is Temperature at the hot side of the thermoelectric module (K); *T*_*C*_ is Temperature at the cold side of the thermoelectric module (K) and *N* is Number of thermocouples in the module.

Leveraging the ocean’s diverse temperatures at varying depths^55^, we maintained a constant warm surface temperature (*T*_*h*_) of 298 K while selecting cold seawater temperatures at progressive 100-m depth intervals. This approach yielded temperature differentials (ΔT) of 6.6 K, 9.8 K, 13 K, 15.7 K, 17.2 K, 18.6 K, 19.8 K, and 20.6 K at a Reynolds number of 11,275. The results revealed a strong correlation between ΔT and system performance.

At the minimum ΔT of 6.6 K, the system generated an open-circuit voltage of 1.09 V, producing a modest output power of 0.304 W with 0.279% conversion efficiency. This relatively low performance stems from the limited thermal gradient available for energy conversion. The reduced temperature differential constrains both the Seebeck voltage generation and the overall thermodynamic efficiency according to Carnot limitations.

In contrast, at the maximum ΔT of 20.6 K, the system achieved significantly better performance with:

- An open-circuit voltage of 3.39 V (210% increase)
- Output power of 2.95 W (870% increase)
- Conversion efficiency of 0.872% (212% increase)

The disproportionate improvement in output power compared to the voltage increase demonstrates the non-linear relationship between voltage and power generation. This non-linearity arises from the quadratic relationship between voltage and power (P = V^2^/R) in electrical systems, where R is given by:

22 $${R}_{TE}={(R}_{n}+{R}_{p})\times N.$$

*R*_*TE*_ is the Total electrical resistance of the thermoelectric module (Ω); Rn is the Electrical resistance of the n-type thermoelectric material (Ω); Rp is the Electrical resistance of the p-type thermoelectric material (Ω).

**Fig. 8:**
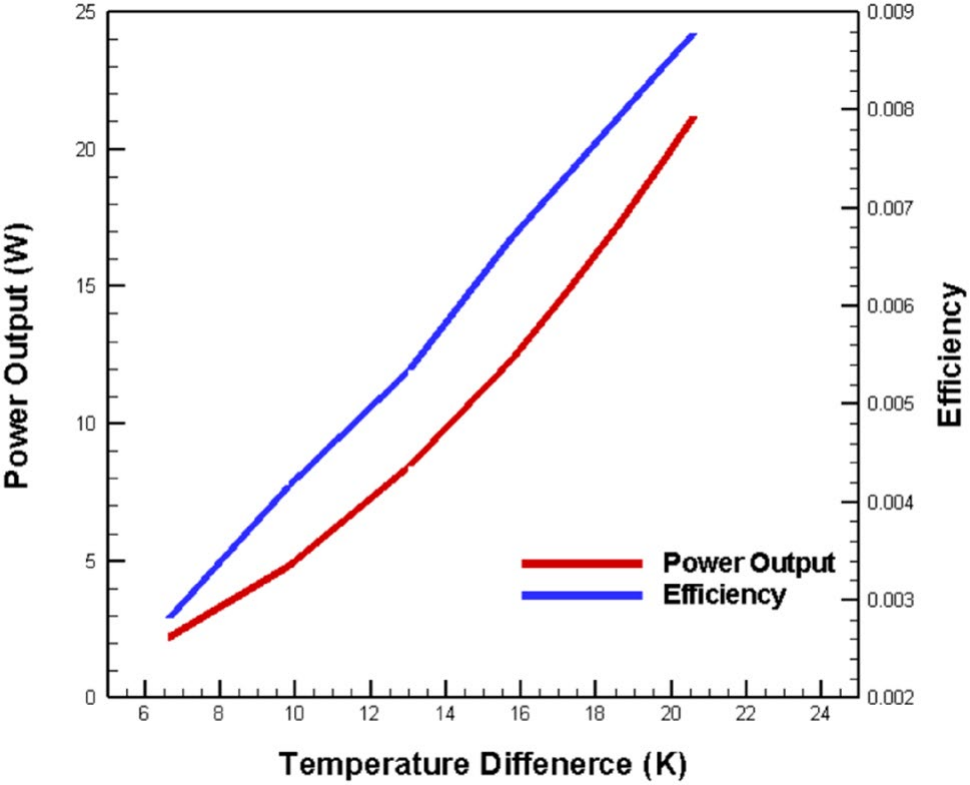
Influence of variations in temperature difference on output power and conversion efficiency^44^.

The data presented in Fig. 8 reveals that both output power and conversion efficiency increase monotonically with temperature differential, but with different scaling behaviors. This difference in scaling can be attributed to the distinct underlying physical mechanisms: while power output benefits directly from increased thermal gradient through enhanced charge carrier movement, efficiency improvements are moderated by increased thermal losses at higher temperature differentials.

These findings have significant implications for OTEC system design and deployment:

- They suggest that optimal system performance requires careful selection of installation depth to maximize the available temperature differential
- The non-linear power scaling indicates that even modest improvements in temperature differential can yield substantial gains in power output
- The efficiency trends provide guidance for cost–benefit analysis when considering deeper (and thus more expensive) cold water pipe installations

This understanding of temperature differential impacts enables more informed decisions about OTEC system placement and design, potentially improving the economic viability of future installations. Compared to earlier work on thermoelectric OTEC by Jayadev et al.^56^, which was largely conceptual, our study provides validated numerical analysis with specific optimization parameters. While they projected 2% theoretical efficiency, we achieved a practical conversion efficiency of 0.872% at ΔT = 20.6 K, supported by detailed flow analysis and experimental validation showing 1.1% error with literature data.

### Integrated internal–external channel flow configuration

This section examines a dual-channel configuration where thermoelectric modules are positioned between inner and outer flow channels. The inner channel carries warm surface seawater while the outer channel utilizes cold deep seawater, creating a thermal gradient across 80 thermoelectric modules along the 0.185 m channel length. This configuration allows for efficient heat transfer between the two fluid streams through the thermoelectric elements.

#### Impact of Reynolds number on pump power consumption, output power, and conversion efficiency

The thermoelectric system is modeled using a square cross-section flow channel. The internal flow channel has a cross-sectional area of 5.63 × 10^–3^ m^2^, while the external flow channel has an area of 1.07 × 10^–1^ m^2^. The internal and external channels are maintained at the same volumetric flow rate, as shown in Table 2. The analysis covers the transition from laminar to turbulent flow, assessing the system’s power output and conversion efficiency. Additionally, the impact of pump power consumption on the thermoelectric system’s power output is evaluated to understand how the pumping requirements influence overall system performance.

**Table 2:** Corresponding Reynolds numbers for varying volumetric flow rates in internal and external channels.

Flow rate (m^3^/s)	${\text{Re}}_{\text{Inner}}$	${\text{Re}}_{\text{Outer}}$
5.625 × 10^–5^	790	73.83
1.125 × 10^–4^	1.581 × 10^3^	147.6
1.638 × 10^–4^	2.3 × 10^3^	215
2.846 × 10^–4^	4 × 10^3^	373.55
3.814 × 10^–3^	5.3628 × 10^4^	5 × 10^4^
7.112 × 10^–3^	1 × 10^5^	9.32808 × 10^3^
8.889 × 10^–3^	1.25 × 10^5^	1.166011 × 10^4^
1.067 × 10^–2^	1.5 × 10^5^	1.400635 × 10^4^
1.244 × 10^–2^	1.75 × 10^5^	1.632415 × 10^4^
5 × 10^–2^	2 × 10^5^	1.86709 × 10^4^

The fluid flow velocity significantly influences heat transfer efficiency. If the flow rate is too low, the heat transfer rate decreases, reducing the heat exchange within the thermoelectric system, resulting in a lower power output. Due to the lower flow velocity at the cold end in the laminar flow regime, the heat exchange is considerably less efficient compared to turbulent flow. Consequently, the laminar flow model’s power output (Pout) and conversion efficiency (η) are much lower.

**Fig. 9:**
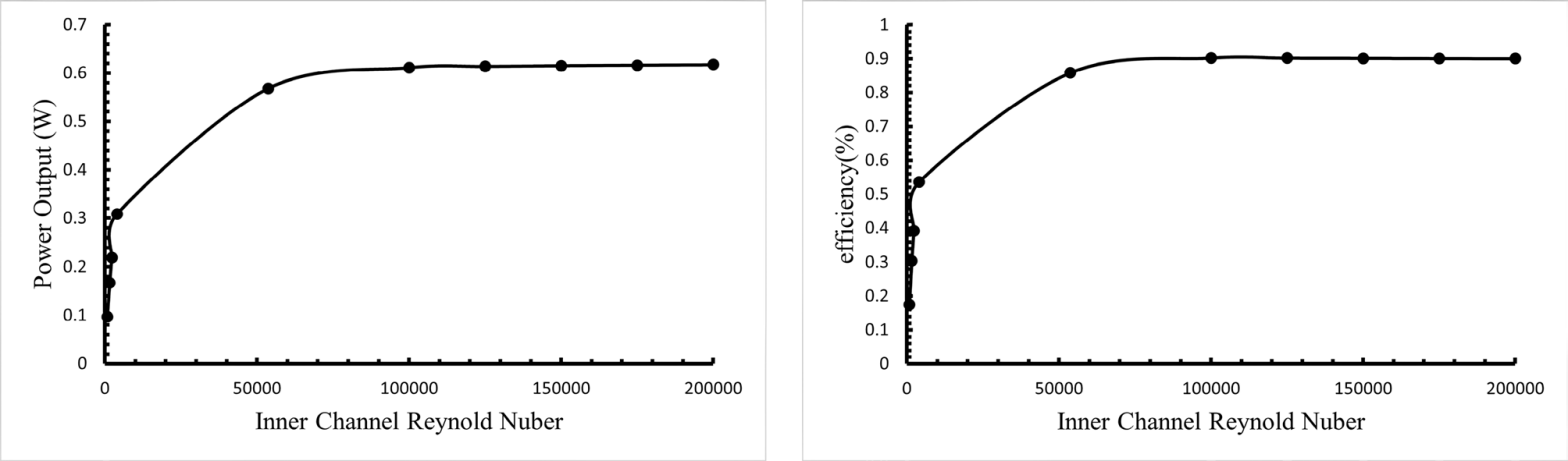
(**a**) Power output, (**b**) efficiency at different Reynolds numbers.

Under turbulent flow conditions, the power output increases with a higher Reynolds number. When the Reynolds number (Re) reaches 50,000 or higher, the maximum power output (P_out_) of 0.61 W is achieved, as shown in Fig. 9a. Similarly, the trend for conversion efficiency follows that of power output, reaching a maximum efficiency (η) of 0.9% at Re ≥ 50,000, as depicted in Fig. 9b.

**Fig. 10:**
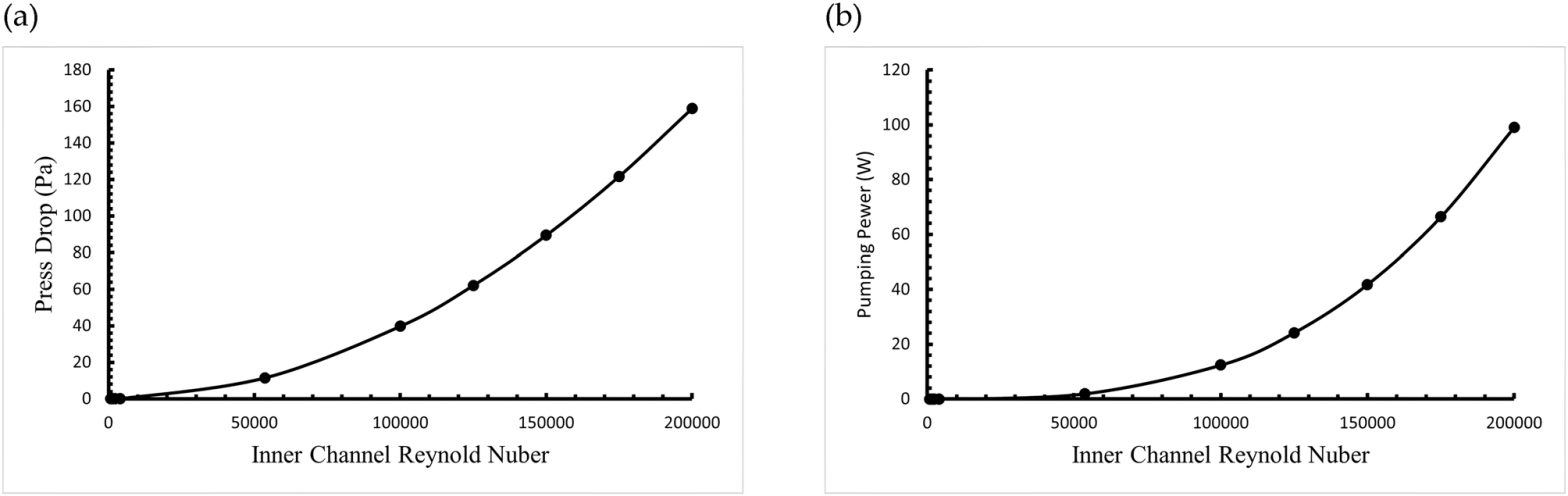
Impact of Reynolds number on (**a**) pressure drop and (**b**) pump power consumption in internal flow channel.

As the fluid flow velocity increases, the pressure drop across the system becomes more pronounced, increasing the power consumption required by the pump. Figure 10a shows the relationship between the pressure at the hot end of the thermoelectric system and the Reynolds number, indicating that the pressure drop increases with flow velocity. As a result, higher flow velocities lead to more significant pressure drops, necessitating more pump power. In contrast, the lower flow velocity under laminar flow conditions results in a smaller pressure drop, reducing pump power consumption.

Figure 10b illustrates the pump power consumption for different Reynolds numbers. While systems with lower Reynolds numbers have lower power output, they require less pump power due to the reduced flow velocity, resulting in a net positive power output. Conversely, at higher Reynolds numbers, the increased flow velocity demands significantly more pump power, which can exceed the power output of the thermoelectric system.

In laminar flow conditions, the net power output increases with the Reynolds number, reaching a maximum of 0.22 W at a Reynolds number of 2300, representing the highest value for laminar flow. However, as the flow transitions into the turbulent regime, the net power output declines and can become harmful. Therefore, when considering pump power consumption, the laminar flow model demonstrates superior power generation efficiency compared to the turbulent flow model.

#### Impact of load resistance to internal resistance ratio on thermoelectric system performance

This study uses a square cross-section flow channel model to analyze the effect of the ratio between load resistance (*R*_*load*_) and internal resistance (*R*_*TE*_) on the thermoelectric system’s power output and conversion efficiency. The internal flow channel has a cross-sectional area of 4 × 10^–2^ m^2^, and the external flow channel has an area of 6.16 × 10^–2^ m^2^. Varying the load resistance evaluates the system’s performance under different practical conditions.

**Fig. 11:**
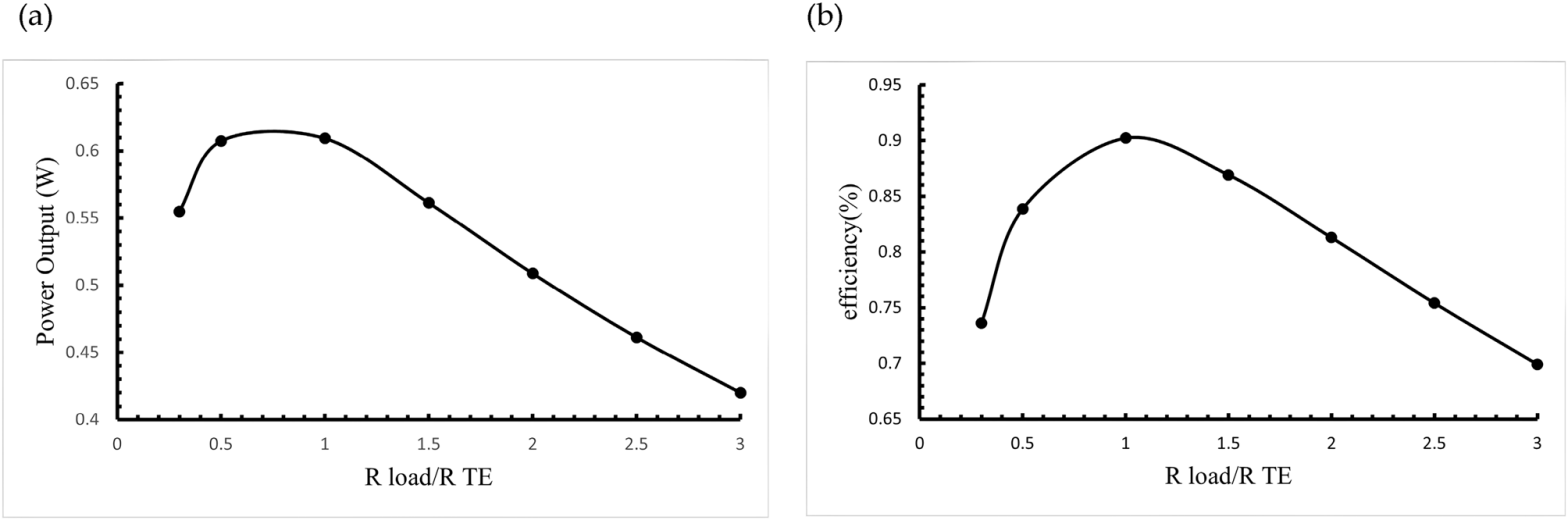
Effect of load resistance/internal resistance ratio on (**a**) output voltage and (**b**) conversion efficiency of the thermoelectric system.

Figure 11 shows the distribution of power output relative to the *R*_*load*_/*R*_*TE*_ ratio and illustrates the corresponding conversion efficiency. The results indicate that when the load resistance is equal to the internal resistance (*R*_*load*_ = *R*_*TE*_), the system achieves maximum voltage, power output, and conversion efficiency.

When the load resistance is less than the internal resistance (*R*_*load*_ < *R*_*TE*_), both power output and efficiency increase as the load resistance increases. Conversely, if the load resistance exceeds the internal resistance (*R*_*load*_ > *R*_*TE*_), the power output decreases as the load resistance rises.

### Case study analysis: system operation under various working conditions

This section integrates the parametric study findings from Sects. 3.1 and 3.2 to establish a practical framework for optimizing system operation under varying environmental conditions. By analyzing key operational parameters, we present a systematic approach to maintaining efficient performance across different temperature differentials, flow conditions, and power demands.

#### Operating conditions analysis

The system achieves optimal performance under tropical deployment conditions, where the temperature differential (∆T) reaches 20.6 K (surface water at 298 K, deep water at 277.4 K). Under these conditions, three key parameters operate in an interdependent manner:

- Channel height: 0.002 m
- Reynolds number: 1.5 × 10^4^
- Net power output: 1.45 W

This operational setting balances heat transfer enhancement and pump power consumption, ensuring stable and efficient energy conversion.

#### System response to temperature differentials

Variations in temperature differentials due to seasonal changes and deployment depths necessitate adjustments in system parameters. Four distinct operational regimes have been identified:

- Optimal Conditions (∆T = 20.6 K):
  - Channel height: 0.002 m
  - Reynolds number: 1.5 × 10^4^
  - Net power output: 1.45 W
  - Efficiency: 0.872%
- High Performance Range (∆T = 13.0 K):
  - Channel height: 0.002 m
  - Reynolds number: 1.5 × 10^4^
  - Net power output: 1.23 W
  - Efficiency: 0.743%
- Moderate Performance Range (∆T = 9.8 K):
  - Channel height: 0.001 m
  - Reynolds number: 1.2 × 10^4^
  - Net power output: 0.82 W
  - Efficiency: 0.521%
- Low Performance Range (∆T = 6.6 K):
  - Channel height: 0.001 m
  - Reynolds number: 1.2 × 10^4^
  - Net power output: 0.45 W
  - Efficiency: 0.279%

The results demonstrate that system performance significantly depends on both temperature differential and channel configuration, with efficiency declining proportionally with decreasing temperature differential.

Based on these operational regimes, we have developed specific strategies to maintain system stability and optimize performance when transitioning between different temperature differential ranges.

#### Dynamic response strategy

To maintain system stability and efficiency under fluctuating environmental conditions, the following adaptive strategies are recommended.

- Adjustments for Temperature Differential Variations:
  For ∆T between 20.6 K and 13.0 K:
    Maintain channel height at 0.002 m
    Keep Reynolds number at 1.5 × 10^4^
    The system maintains 85% of optimal power output (1.23 W)
  For ∆T below 13.0 K:
    Reduce channel height to 0.001 m
    Adjust the Reynolds number to 1.2 × 10^4^
    Power output reduces to 0.82 W at ∆T = 9.8 K
- Material Performance Optimization:
  The Bi₂Se₀.₅Te₂.₅ (n-type) and (Bi₀.₂Sb₀.₈)₂Te₃ (p-type) combination demonstrates:
    1.0 W higher output at ∆T = 20.6 K
    0.4 W advantage at ∆T = 6.6 K
    Superior performance across all temperature ranges
- Pump Power Management:
  Optimal net power (1.45 W) is achieved at a channel height of 0.002 m
  Net power output becomes negative when channel height exceeds 0.01 m due to pump power consumption
  Channel height reduction provides better system efficiency by minimizing pump power requirements

#### System limitations and operational boundaries

The parametric studies reveal several critical operational limits:

- Temperature Differential Constraints:
  Minimum viable ∆T: 6.6 K
  Maximum achievable ∆T: 20.6 K
- Channel Height Constraints:
  Minimum: 0.001 m (limited by manufacturing and maintenance capabilities)
  Maximum: 0.002 m (limited by pump power efficiency)
- Flow Rate Boundaries:
  Minimum Reynolds number: 1.1 × 10^4^ (required for adequate heat transfer)
  Maximum Reynolds number: 1.5 × 10^4^ (limited by pump power consumption)

These operational guidelines provide a comprehensive framework for maintaining optimal system performance while adapting to varying environmental conditions in practical OTEC deployments.

### Techno-economic assessment

This section presents a comprehensive techno-economic analysis of the proposed Bi₂Te₃-based thermoelectric OTEC system, evaluating its commercial viability through systematic cost modeling and market competitiveness assessment methodologies.

Scaling Methodology: While earlier conceptual models assessed a 400 MWe scale^56^, this study focuses on scalable designs based on 10 and 100 MWe systems for practical deployment analysis. The techno-economic analysis scales the laboratory-validated simulation results to commercial deployment scenarios. The simulation demonstrated a net power of 1.45 W per 400-unit TEG module under optimal conditions (0.002 m channel height, Re > 12,000). For commercial viability assessment, we analyze 10 MWe demonstration and 100 MWe commercial systems, assuming optimized modules that achieve 5 W of net power each through anticipated improvements in manufacturing processes, enhanced thermal interface designs, and superior material integration, as identified in Section "Performance Integration:" optimization studies. This scaling approach maintains technical accuracy while providing realistic commercial projections.

### Economic viability analysis

#### Capital expenditure assessment

The capital cost analysis examines two deployment scenarios: a demonstration-scale 10 MWe system and a commercial-scale 100 MWe system. Table 3 presents a detailed breakdown of capital costs, highlighting the dominance of thermoelectric materials in the overall investment.

**Table 3:** Capital cost breakdown for scaled thermoelectric OTEC systems.

Component category	Item	10 MWe system	100 MWe system	Unit basis
Thermoelectric materials	Bi₂Te₃-based semiconductors	168–240 M	1,680–2,400 M	280–400/kg, 0.3 kg/module
	Electrical contacts (Cu)	2.1 M	21 M	10.30/kg (current market)
	TEG modules (total)	2 × 10⁶ units	20 × 10⁶ units	5W net per module
Heat exchange system	Cu–Ni alloy (90/10)	39 M	325 M	9.80/kg
	Heat exchanger fabrication	16 M	135 M	Assembly costs
Seawater circulation	Marine pumps	12 M	85 M	1000–10,000 m^3^/s capacity
	Piping system	15 M	120 M	Cold water intake
Infrastructure	Offshore platform	55 M	385 M	Scale factor 0.7
	Mooring system	28 M	195 M	Depth and size dependent
	Power transmission	35 M	640 M	Grid connection
Total capital cost		370–442 M	3586–4306 M	
Specific cost		37.0–44.2 M/MW	35.9–43.1 M/MW	Scale benefits

The analysis reveals that thermoelectric materials account for 45–54% of the total capital costs for the 10 MWe system and 47–56% for the 100 MWe system, representing the primary target for cost reduction. Updated pricing reflects current market conditions based on 2024 market analysis: Bi₂Te₃ at USD 280–400/kg and copper at USD 10.30/kg. The scaling analysis demonstrates modest economies of scale, with specific costs reducing from USD 37.0–44.2 million/MW for the 10 MWe system to USD 35.9–43.1 million/MW for the 100 MWe system.

Scaling Validation: The cost estimates use verified market prices and realistic module requirements derived from simulation results. The 10 MWe system requires 2 million TEG modules (each producing 5 W of net power), while the 100 MWe system requires 20 million modules. This accounts for current thermoelectric material costs and bulk procurement considerations.

#### Operational cost structure and performance metrics

The operational economics demonstrate favorable characteristics for marine energy applications, with performance metrics reflecting the inherent advantages of thermoelectric systems in harsh ocean environments. Table 4 presents key operational parameters that directly impact the long-term economic viability and competitiveness of the system.

**Table 4:** Operational performance and cost analysis.

Parameter	10 MWe system	100 MWe SYSTEM	Units	Benchmark comparison
Performance metrics				
Net power output	10	100	MWe	Demonstration vs. commercial scale
Capacity factor	85–90	85–90	%	Superior to solar (25%) and wind (35%)
System lifetime	25	30	years	Marine infrastructure standard
Annual energy production	74–79	744–789	GWh	6,900–73,000 households
Operating costs				
Annual maintenance	3.5	3.0	% of CAPEX	Expected to improve with system scale
TEG module replacement	7% every 5	5% every 5	years	Expected to improve with system scale
Heat exchanger replacement	5% every 10	5% every 10	years	Standard marine equipment
Biofouling prevention	0.5	2.5	Million USD/year	Scale-dependent treatment

The high capacity factor of 85–90% represents a significant operational advantage over intermittent renewable sources, providing consistent baseload power generation that enhances grid stability and reduces backup power requirements. This reliability factor is particularly valuable for island applications where energy security is paramount.

The operational analysis reveals several key advantages of thermoelectric systems over conventional OTEC designs, highlighting the inherent benefits of solid-state energy conversion. The absence of working fluid circulation eliminates risks associated with ammonia leakage and reduces system complexity. Maintenance requirements are significantly lower due to the solid-state nature of thermoelectric conversion, with no moving parts in the primary energy conversion system. The modular design of TEG arrays allows for partial system operation during maintenance, maintaining power output even when individual modules require service.

System management represents a smaller operational burden compared to conventional OTEC systems due to the reduced mechanical complexity of thermoelectric systems. The direct thermal contact design eliminates the need for intermediate heat transfer fluids, simplifying maintenance procedures and reducing the potential for system downtime. These operational characteristics translate to improved system availability and reduced lifecycle maintenance costs, factors that are particularly valuable in remote marine deployments where maintenance access is limited and expensive.

#### Levelized cost of energy analysis

The LCOE analysis incorporates both current market conditions and projected cost reduction scenarios, with realistic scaling from the simulation-validated performance parameters. The analysis shows a clear pathway from demonstration to commercial viability through systematic cost reduction strategies.

Current LCOE Assessment reveals that the 10 MWe demonstration system achieves $0.32–0.42/kWh, while the 100 MWe commercial system shows improvement to $0.25–0.35/kWh. These costs reflect the premium associated with thermoelectric technology compared to conventional OTEC systems ($0.15–0.30/kWh), but remain competitive with remote island electricity costs that typically range from $0.30–0.50/kWh for diesel generation. The primary cost drivers include material costs representing 50% of LCOE, capital recovery accounting for 25%, operations and maintenance contributing 15%, and component replacement costs comprising the remaining 10%. This cost structure reflects the material-intensive nature of thermoelectric technology and highlights the critical importance of material cost reduction strategies.

Cost Reduction Pathway Analysis demonstrates that projected LCOE improvement to $0.20–0.28/kWh for the 100 MWe system depends on three critical advancement areas working synergistically. Material cost reduction offers the greatest potential savings of 35% through advanced manufacturing processes that reduce material waste by 25%, alternative material formulations that maintain 90% of current performance while reducing costs, and economy of scale effects that could reduce material costs by 20–30% through bulk procurement and optimized supply chains.

Manufacturing optimization represents the second major cost reduction opportunity, targeting 25% assembly cost reduction through multiple pathways. Automated assembly processes could reduce labor costs by 40% while improving consistency and quality. Improved module integration techniques have the potential to increase manufacturing yield by 15%, reducing waste and rework costs. Standardized component designs could reduce customization costs by 30% through economies of scale in production and simplified assembly procedures.

Design efficiency improvements constitute the third major opportunity, resulting in a 20% improvement in material utilization efficiency through targeted engineering solutions. Optimized thermal interface designs could enhance heat transfer by 15%, enabling smaller systems to achieve equivalent power output. Advanced channel configurations based on the simulation findings from Section "Performance Integration:" offer opportunities for improved performance without proportional cost increases. Parasitic losses can be minimized through improved system integration, particularly pump power optimization, as demonstrated in the simulation results, which could substantially improve net power output and overall system performance.

Market Competitiveness Analysis indicates that while current LCOE remains higher than conventional power sources, the technology shows strong promise for niche applications. Remote islands with electricity costs exceeding $0.40/kWh represent immediate market opportunities where the technology’s reliability and baseload characteristics provide compelling value propositions despite higher initial costs.

### Market competitiveness and deployment strategy

#### Comparative economic analysis

The market competitiveness analysis positions the thermoelectric OTEC technology within the broader renewable energy landscape. Table 5 provides a comprehensive comparison with competing technologies for similar deployment scenarios.

**Table 5:** Technology comparison for island and remote coastal applications.

Technology	LCOE ($/kWh)	Capacity factor (%)	Technical maturity
Proposed TE-OTEC	0.20–0.28	85–90	Demonstration
Conventional OTEC	0.15–0.20	90–95	Commercial
Offshore wind	0.08–0.12	35–45	Commercial
Diesel generation	0.25–0.40	90–95	Mature
Solar + storage	0.20–0.30	25–30	Commercial

The analysis reveals that while the current LCOE is higher than some alternatives, the combination of high capacity factor and potential for cost reduction creates a compelling value proposition for specific market segments.

#### Market penetration strategy and timeline

The market deployment strategy identifies three distinct phases aligned with technology maturation and cost reduction achievements, progressing from niche applications to mainstream integration over a 15-year timeline.

Phase 1 (2025–2030): Niche Market Entry represents the initial commercial deployment contingent upon successful pilot validation and supportive policy frameworks. This phase would focus on remote islands with high electricity costs exceeding USD 0.35/kWh, emphasizing demonstration systems ranging from 5–20 MWe, where the technology’s high reliability and baseload characteristics provide clear value despite higher costs. Key success factors include operational reliability demonstration and supply chain establishment, with an estimated market size of USD 2–5 billion supporting approximately 10–15 systems globally. The focus during this phase centers on proving commercial viability in favorable economic environments where conventional alternatives are limited or expensive.

Phase 2 (2030–2035): Regional Market Expansion builds upon demonstrated reliability to target coastal regions with favorable thermal gradients. Deployment scales increase to 50–100 MWe commercial systems as manufacturing economies of scale reduce costs to approximately USD 0.25/kWh. This phase requires proven commercial operation and established supply chains, targeting a market size of USD 15–25 billion with an estimated 25–40 systems globally. Regional expansion focuses on areas where thermal gradients, grid infrastructure, and regulatory frameworks align to support larger installations.

Phase 3 (2035–2040): Specialized Integration represents full market maturity with grid-scale renewable energy portfolio integration. Deployment scales reach 100 + MWe utility-scale systems as the technology achieves grid parity and benefits from supportive policy mechanisms. This phase targets a market size of USD 25–50 billion, supporting an estimated 50–100 systems globally. Success depends on achieving cost competitiveness with other renewable technologies while leveraging the unique advantage of consistent baseload power generation that thermoelectric OTEC systems provide.

#### Risk assessment and mitigation strategies

The comprehensive risk analysis identifies key technical, economic, and regulatory challenges that could impact commercial deployment. Table 6 summarizes the primary risk factors and corresponding mitigation strategies.

**Table 6:** Risk assessment and mitigation framework.

Risk category	Risk factor	Probability	Impact	Mitigation strategy
Technical	Material degradation	Medium	High	Accelerated testing, redundant design
	Biofouling	High	Medium	Advanced antifouling, maintenance protocols
	System integration	Medium	Medium	Modular design, proven interfaces
Economic	Material cost volatility	High	High	Long-term supply contracts, alternative materials
	Currency fluctuation	Medium	Medium	Financial hedging, local sourcing
	Competition from alternatives	High	Medium	Continuous innovation, niche focus
Regulatory	Environmental permits	Medium	High	Early stakeholder engagement, compliance design
	Grid interconnection	Low	High	Utility partnerships, technical standards
	International deployment	Medium	Medium	Local partnerships, technology transfer

### Study limitations and uncertainties

This techno-economic analysis is subject to several important limitations that should be considered when interpreting the results.

#### Technology maturity limitations

Laboratory-to-Commercial Gap: The performance projections are based on laboratory-scale thermoelectric achievements that have not been demonstrated at commercial scale. The 5W net power per module assumes successful scaling of the current 1.45W laboratory results, which may encounter unforeseen engineering challenges.

Manufacturing Readiness: The cost reduction assumptions for mass production (35% material cost reduction, 25% assembly cost reduction) are projections not validated by actual industrial-scale thermoelectric manufacturing experience. Current thermoelectric production is primarily for niche applications at much smaller scales.

Long-term Reliability: The 25–30 year system lifetime assumption lacks validation from long-term operational data of thermoelectric systems in marine environments. Most existing data comes from shorter-term laboratory studies or terrestrial applications.

#### Economic model limitations

Cost Competitiveness Gap: The projected LCOE of USD 0.25–0.35/kWh for 100 MWe systems remains 5–11 times higher than current utility-scale solar (USD 0.044/kWh) and wind (USD 0.033/kWh) costs, limiting market competitiveness to specialized applications where baseload reliability justifies premium costs.

Material Price Volatility: Bi₂Te₃ costs (USD 280–400/kg) are subject to significant supply chain constraints and price volatility. Large-scale deployment could face material availability bottlenecks, potentially driving costs higher than projected.

Learning Curve Assumptions: The cost reduction pathways assume technological learning rates similar to other renewable technologies, but thermoelectric OTEC represents a fundamentally different technology with potentially different learning dynamics.

#### Market and deployment constraints

Limited Addressable Market: The technology appears economically viable primarily for remote applications with electricity costs exceeding USD 0.35/kWh, representing a small fraction of global electricity demand.

Supply Chain Dependencies: The analysis assumes development of a mature supply chain for specialized thermoelectric components without accounting for the time and investment required to establish such infrastructure.

Regulatory and Environmental Uncertainties: Marine deployment faces uncertain regulatory frameworks and potential environmental impact assessments that could affect project timelines and costs.

#### Technical performance limitations

Simulation vs. Real-World Performance: The performance calculations are based on idealized simulation conditions that may not be achievable in actual marine environments with biofouling, variable ocean conditions, and system degradation.

Heat Transfer Assumptions: The assumed heat transfer coefficients and thermal resistance values may not account for real-world factors such as marine biofouling, corrosion, and long-term degradation in seawater environments.

Parasitic Load Uncertainties: Pumping power calculations assume optimal system design and may underestimate real-world parasitic losses from control systems, auxiliary equipment, and operational inefficiencies.

#### Appropriate use of this analysis

This study should be interpreted as:

- Order-of-magnitude assessment suitable for research planning and technology roadmap development
- Baseline analysis for identifying key technical and economic challenges requiring further investigation
- Comparative framework for evaluating thermoelectric OTEC against other ocean energy technologies

This analysis should not be used as:

- Definitive cost estimates for commercial project development without additional validation
- Investment-grade feasibility studies without site-specific and market analysis
- Broad market deployment projections without considering competing technology improvements

### Discussion and implications

The comprehensive techno-economic analysis confirms that Bi₂Te₃-based thermoelectric OTEC systems offer a viable pathway for sustainable ocean energy conversion in specific market segments. While current capital costs remain substantial (USD 370–442 million for 10 MWe), the technology demonstrates clear value propositions for remote island applications and specialized energy requirements where conventional alternatives are limited or expensive.

The analysis establishes two key findings that position thermoelectric OTEC as a promising complement to the renewable energy portfolio in niche applications. First, the economic viability pathway shows potential for commercial competitiveness in specialized markets through systematic cost reduction, with LCOE improving from USD 0.32–0.42/kWh to projected USD 0.20–0.28/kWh through material innovation and manufacturing optimization. Second, the strategic market positioning reveals a clear deployment pathway progressing from niche applications to specialized integration as technology matures and costs decline.

The technology’s primary competitive advantage lies in its ability to provide consistent baseload power with high capacity factors (85–90%), addressing a critical gap in renewable energy portfolios dominated by intermittent sources. While the LCOE remains above mainstream renewables, the reliability premium may justify deployment in applications where energy security and grid stability are prioritized over minimum cost.

Continued innovations in materials science, manufacturing automation, and system integration will be critical in bringing these systems to commercial maturity and realizing their potential for reliable baseload renewable energy generation in specialized applications.

## Conclusions

This research thoroughly investigates the integration of Bi₂Te₃-based thermoelectric generators (TEGs) in Ocean Thermal Energy Conversion (OTEC) systems through detailed finite element simulations. The quantitative analysis reveals optimal configurations and comparative performance metrics, with specific numerical results for each key parameter:

1. Flow Configuration and Channel Design:
  Reynolds numbers above 12,000 ensure stable heat supply to TEGs, resulting in consistent output power of 3.01 W.
  Optimal channel height of 0.002 m yields a maximum net power of 1.45 W.
  Critical threshold identified at 0.01 m channel height, above which net power becomes negative due to pump power requirements.
  Among all tested configurations, the optimal TEG-OTEC system combines a 0.002 m channel height operating above Reynolds number 12,000, maximizing net power output while minimizing pump power consumption. This configuration is superior to both higher channel heights and lower Reynolds number flows, ensuring stable heat exchange with minimal energy loss.
2. Temperature Differential Impact:
  Maximum performance achieved at ΔT = 20.6 K:
    Power output: 2.95 W
    Conversion efficiency: 0.872%
  Minimum Tested differential ΔT = 6.6 K:
    Power output: 0.304 W
    Conversion efficiency: 0.279%
  This study highlights that optimizing temperature differential selection is crucial for balancing power output and efficiency. The observed trends suggest that practical TEG-OTEC implementation should target ocean depths with stable thermal gradients, ensuring consistent energy generation in offshore environments.
3. Flow Regime Performance:
  Turbulent flow (Re ≥ 50,000):
    Maximum power output: 0.61 W
    Peak conversion efficiency: 0.9%
  Laminar flow:
    Best performance at Re = 2300
    Maximum net power: 0.22 W
  The results underscore that while turbulent flow maximizes power generation, laminar conditions offer better energy efficiency due to lower pump power consumption. These findings highlight the need to optimize flow conditions based on specific operational constraints, such as system scale and deployment location.
4. Material Performance:
  Bi₂Se₀.₅Te₂.₅ (n-type) with (Bi₀.₂Sb₀.₈)₂Te₃ (p-type) showed approximately 1 W higher output power compared to the 75% Bi₂Te₃ + 25% Bi₂Se₃ (n-type) and 25% Bi₂Te₃ + 75% Sb₂Te₃ (p-type)
  Improved conversion efficiency by 0.5% in counter-current configuration.

This analysis confirms that selecting thermoelectric materials with higher electrical conductivity and lower thermal conductivity significantly enhances OTEC system performance. Future material innovations focusing on these properties could further improve energy conversion efficiency in marine applications.

This study identifies the optimal TEG-OTEC system configuration as a 0.002 m channel height with Reynolds number above 12,000, ensuring maximum net power output while minimizing pump power consumption. These findings provide essential design guidelines for developing practical TEG-OTEC systems, particularly for offshore applications where operational efficiency and system durability are critical. These quantitative findings establish clear design parameters and performance benchmarks for implementing TEG-OTEC systems in marine environments, contributing to the advancement of sustainable ocean energy technologies.

## Data Availability

Data can be obtained by contacting the author, Chun-I Wu (wuchuni@ntou.edu.tw).
